# Climate Change, Water Quality and Water-Related Challenges: A Review with Focus on Pakistan

**DOI:** 10.3390/ijerph17228518

**Published:** 2020-11-17

**Authors:** Toqeer Ahmed, Mohammad Zounemat-Kermani, Miklas Scholz

**Affiliations:** 1 Centre for Climate Research and Development, COMSATS University Islamabad, Park Road, Chak Shahzad, Islamabad 45550, Pakistan; toqeer.ahmed@comsats.edu.pk; 2 Department of Water Engineering, Shahid Bahonar University of Kerman, Kerman 7616913439, Iran; mohammad.zounemat@gmail.com; 3 Division of Water Resources Engineering, Faculty of Engineering, Lund University, PO Box 118, 22100 Lund, Sweden; 4 Department of Civil Engineering Science, School of Civil Engineering and the Built Environment, University of Johannesburg, Kingsway Campus, Aukland Park 2006, Johannesburg PO Box 524, South Africa; 5 Civil Engineering Research Group, School of Computing, Science and Engineering, The University of Salford, Newton Building, Peel Park Campus, Salford M5 4WT, UK

**Keywords:** climate variability, developing country, health impact, water and vector-borne disease, water pollution and policy, water resources management

## Abstract

Climate variability is heavily impacting human health all around the globe, in particular, on residents of developing countries. Impacts on surface water and groundwater resources and water-related illnesses are increasing, especially under changing climate scenarios such as diversity in rainfall patterns, increasing temperature, flash floods, severe droughts, heatwaves and heavy precipitation. Emerging water-related diseases such as dengue fever and chikungunya are reappearing and impacting on the life of the deprived; as such, the provision of safe water and health care is in great demand in developing countries to combat the spread of infectious diseases. Government, academia and private water bodies are conducting water quality surveys and providing health care facilities, but there is still a need to improve the present strategies concerning water treatment and management, as well as governance. In this review paper, climate change pattern and risks associated with water-related diseases in developing countries, with particular focus on Pakistan, and novel methods for controlling both waterborne and water-related diseases are discussed. This study is important for public health care, particularly in developing countries, for policy makers, and researchers working in the area of climate change, water quality and risk assessment.

## 1. Introduction

Climate variability involving changes in temperature, rainfall pattern and precipitation is increasing and heavily impacting on water resources, water-related diseases and, subsequently, human health, which is reliant on clean water. Water-related infectious diseases like malaria, dengue fever, chikungunya, along with their causative agents and the mode of transmission of these diseases have been affected by climate variability. Similarly, waterborne diseases like typhoid and cholera are influenced by climate change patterns, and subsequent risks related to these diseases are increasing [1,2,3,4]. About five cases of dengue develop into a hemorrhagic fever from 390 million dengue fever infections around the globe [5]. In order to save people from disaster, it has been suggested that poor and developing countries need to save, grow, invest, and protect poor and vulnerable people from economic crises [6]. Adaptation strategies related to these changes are important for policy and impact assessments [7].

Globally, almost all countries are affected by climate change impacts, particularly the developing countries, which are more vulnerable and prone to disasters like extreme floods, droughts, storms and heatwaves. In the last decade, a decline in economic growth has been observed in some developing countries, and people living in these countries are most affected as they do not have the resources to cope with the occurring natural disasters [8,9]. Half of the world’s poor population lives in Sub-Saharan Africa. Significant poverty reductions have been observed in East Asia, especially China and Indonesia, between 2012 and 2013 [6]. However, developing countries are still suffering from economic problems, especially people living in rural agricultural areas with no access to essential resources in order to gain education [6,10].

Roser and Ortiz-Ospina [11] reported that people earning less than 3.10 US $/day (less than Pak Rs (PKR). 500) mostly live in countries such as Pakistan, India, Bangladesh and Ethiopia. Poor people are more vulnerable to natural disasters. Pakistan is at number 7 in a list of endangered countries, with 70% of its population exposed to natural hazards [12]. Millions of people in India and Bangladesh are exposed to floods. Due to climate variability, even developed countries like Japan, Hong Kong and Taiwan have been exposed to at least one type of natural hazard in the past few years. The global Climate Risk Index indicates the extent of vulnerability of a country from weather-related events like flooding, drought, heat waves and storms [13]. A low climate risk index (CRI) value indicates the highest vulnerability as some countries are more prone to frequent disaster. Of the top ten most affected countries by natural disasters, nine were from developing countries with low-middle-income, all except for Thailand (Table 1). Among them, Serbia, Afghanistan and Bosnia and Herzegovina were the most affected [14]. Pakistan and the Philippines are affected recurrently by catastrophes. They are commonly ranked among the most affected countries. According to CRI 2018, the Philippines are the second most affected country among the top ten climate change-affected countries. The CRI [10] indicated that Pakistan was at number eight in the list of most affected countries between 1995 and 2014 (Table 1) [10].

More than 2.5 billion individuals (30% of the world’s residents) are at risk of dengue fever, particularly in Southeast Asia, the Americas, and the Western Pacific. According to the UN water report [15], world water demand will increase by up to 55% by 2050 due to more demand by industry, domestic consumption, food production and electric generation use. Similarly, global demand for food will increase by 60% (100% in the developing countries) by 2050 due to an increase in population [15]. Stress on sustainable water management will increase due to poverty, unequal distribution of resources, inequitable access to resources and poor management.

The current situation indicates that mitigation and improved adaptation strategies are required to minimize the impacts of climate variability. This study analyzes recent scenarios impacted on by population increase, water-related disasters, water pollution and how to control diseases linked to water. The main objectives of this paper are to analyze climate variability and water-related disasters as well as their impacts on human health. Finally, some key recommendations are made for policy-makers.

## 2. Methodology and Review

### 2.1. Literature Selection

In this study, the authors assessed peer reviewed research papers, reports and grey literature published after 1979. Websites including google scholar (https://scholar.google.com.pk), Web of Knowledge (http://isiknowledge.com), ScienceDirect (http://www.sciencedirect.com) and Scopus (https://www.scopus.com) were searched for relevant literature. More attention has been paid to recent but already well-referenced literature. Relevant literature was selected based predominantly on the following inclusion criteria: (a) peer-reviewed research papers published by impact factor-listed research journals; (b) peer-reviewed scientific reports from world-known publishers; (c) literature was screened by using keywords (climate variability; climate and water quality; waterborne; water-related disease; dengue fever and health impacts; Zika virus; Chikungunya; method for controlling waterborne diseases; temperature and precipitation effects; developing countries; population and water quality; climate change impacts on chemical water quality; water quality in Pakistan; water governance; water management; and water pollution); and (d) preference was given to studies published in English language.

### 2.2. Climate Variability

Climate variability is a growing concern worldwide [16]. Climate change deeply impacts on social and natural environments and is one of the major threats to public health [17,18]. The water quality of recreational waterbodies such as coastal waters is considerably affected by extreme weather conditions like storms and typhoons, which increase the contamination of drinking water leading to water-borne diseases [19].

Changes in climate have varied greatly and influenced water resources, groundwater contamination, health and subsequently human life [20,21]. High uncertainty regarding expected changes in temperature and rainfall in the upcoming years has been reported in some studies [22]. It has been estimated that the average global temperature for the last hundred years has increased overall by approximately 0.8 °C due to the emission of greenhouse gases, and recent years were announced as the hottest in recent history. Due to the increase in global temperature, changes in precipitation levels have not been uniform in recent decades. As a result, monsoon rainfalls are more likely to happen in humid and sub-humid areas, whereas there will be a decrease in winter and summer rainfalls in coastal and hyper-arid areas. Besides, it has been claimed that sea levels will rise to a range of 1 to 3 mm per year [23,24]. There is also uncertainty about rainfalls with uneven temporal and spatial distribution, and longer dry spells evoking drought conditions [25].

Indeed, due to human activities, the mean temperature on the surface of the earth has been increasing over the past century [26]. It has been estimated that hot summer days have also become more extended and regular in some parts of the globe. Increased surface temperature is leading to an increase in evaporation from the oceans and land. Accordingly, there will be an increase in global average precipitation. Some regions also experience droughts due to high evaporation levels and shifting of wind patterns while some parts of the world receive flash floods. However, it is very difficult to differentiate whether an extreme weather event is caused by natural or human influences [27]. In a study by Levy et al. [28], the general effects of climate change on water-borne diseases have been investigated. Other studies have focused on specific components of climate change such as the impact of short-term extreme flood events on infectious diseases [20,29].

Global warming causes the temperature to rise and, as a result, low-level glaciers are melting [30]. About 76 lakes covering an average area of 545 ha in high mountainous regions were studied. Regular monitoring of glaciers was recommended to support water management in the context of climate variability [31]. Temperature may increase this century by 2%–6 °C, which will particularly impact negatively on water resources in Central Asia which depend commonly on river water for agriculture [32].

Glaciers are one of the most important sources of water for Asian countries. About 41% of the area of glaciers are vulnerable to climate change in China [33]. Climate change is linked to an increase in mean temperature [23] and is the main factor in the melting of glaciers [34]. This has also led to changes in precipitation pattern, diversity and rate. Since 1900, changes in precipitation patterns amounted to an approximately 2% increase over the land area of the globe [35,36]. Likewise, a correlation between the increase in streamflow and precipitation has been identified [37,38,39].

It was reported that roughly 80% of diseases in developing countries such as Pakistan are related to waterborne diseases [40]. In Pakistan, water quality is being impacted by climate change through temperature and rainfall fluctuations [41]. A study showed that the maximum temperature has significantly augmented (in over 30% of sites) during the pre-monsoon season annually [42]. A considerable increase was observed in March. The minimum temperature showed positive trends for the pre-monsoon season at the annual scale. There was a cooling trend in the northern areas during the study period. The maximum temperature increased faster than the minimum temperature in the northern areas during all seasons studied and at annual resolution, while the opposite occurred for the rest of the country (except during the pre-monsoon season). It has been estimated that the highest correlation coefficients between patterns and both minimum and maximum temperatures were observed in the months of the pre-monsoon season [43].

### 2.3. Water Pollution, Population and Water Quality

The world population is expanding, with a total of 7.4 billion in 2016, and is expected to increase in the upcoming decades [44]. The eight most populous countries have a combined population of over 4.054 billion, which is expected to increase to 4.980 billion by 2050 (Table 2). With this increase in population, water resources are under stress, especially in the developing countries.

Water pollution is directly related to population growth and has a direct impact on human health. Population growth and anthropogenic activities heavily influence water resources. The demand for water is augmented along with an increase of population, and ultimately the quality of water resources will be affected [45]. According to data for the world’s most water-stressed countries [46], Pakistan is among the most vulnerable, and will become a water-stressed country by 2040 [47,48].

According to Vineis et al. [49], about 884 million people are living without access to clean drinking water in 2019. Poor quality of water, especially drinking water, increases the chances of waterborne diseases [40]. About 1.8 million people die every year due to cholera and diarrhea, and 3900 children die every day due to poor water and sanitation conditions [50]. Similarly, more than one billion people lack access to improved drinking water, particularly those living in Asia [51]. In developing countries, the population is increasing, and cities will be overpopulated in the next 20 years. Accordingly, demand for improved water resources management, water quality control and enhanced flood and drought management will increase [52].

As reported by the WHO [53], half of the world’s population will suffer water stress conditions by 2025. Similarly, along with water shortage, water quality is also negatively affected, so that 1.8 billion people around the world are obliged to consume water contaminated by sewerage for drinking, which practice transfers diseases like cholera, typhoid, dysentery and polio. Empirical studies have already indicated the downside effects on human health of pollution and poor water quality due to the rapid increase in population and urbanization [54]. Regions or countries facing climate challenges and natural disasters such as drought and floods have also to endure population growth problems, and inevitably anthropogenic activities alter water systems [55]. A decrease in water resources due to less income and slow development will increase the problems of water quality and health issues. Water availability has been decreasing in all sectors by 7–11% during the last two decades [41]. Water availability is affected by climate change as well as water governance and management issues. There is a need to increase water storage capacity and installation of water retention wells for groundwater recharge. Groundwater regulations have been approved by all provinces of Pakistan except for Sindh, but implementation of polices in the true sense are lacking. By area, Sindh is the third largest province of Pakistan and by population the second largest. This is important as Karachi city (the former capital) is the largest city of Sindh province. Incentives should be implemented for the general public to obey governmental rules for water saving and fines imposed on violators. The government should implement licensing for the installation of new bore wells and there should be a record of the number of tube and bore wells installed, as no such data exist especially for private bore wells.

Water quality is linked with water availability. Water quality analysis of the major cities of Pakistan has been recently completed by the government. Similarly, other research and development organizations and non-governmental organizations (NGO) are performing water quality analysis especially in rural areas. Bacteriological water quality is often more important than chemical water quality as water resources are contaminated with fecal matter. No data on gastroenteritis have been found in allied hospitals when asked for records of patients suffering from food or waterborne diseases. It is strongly recommended in hospitals that records of people suffering from waterborne diseases are maintained.

### 2.4. Climate, Water-Related Diseases, and Health Impacts

Climate variability effects climate-sensitive diseases like dengue fever, diarrhea and cholera [56,57,58,59]. Microclimatic parameters, especially precipitation and temperature, play a key role in spreading waterborne and water-related diseases [60,61,62,63,64]. Microbiological, bioinformatics and genomic tools have provided some evidence that El Niño is the main key element in triggering long distance spread of cholera [65]. Climate change has a direct effect on the reemergence of waterborne infectious diseases such as cholera [66]. It is expected that diarrhea rates will be aggravated in many developing countries due to changes in climate, but the extent will vary depending on the nature of change, region and local climate [67,68]. A direct relation has been observed between climate-related disasters such as floods, heavy rainfalls and waterborne diseases. Typically, waterborne diseases and zoonotic infections increase after floods and rainfall, and high temperature also supports the growth of waterborne diseases [69]. There is a correlation between waterborne diseases and wet summer and humid weather. Typhoid is linked to dry weather in Europe [70]. Climate change could also pose an increased health risk linked to pathogens like *Campylobacter, Cryptosporidium* and norovirus. Norovirus and *Cryptosporidium* are less temperature-sensitive and are more resilient than *Campylobacter* [71]. Legionella species are ubiquitous in natural settings, share common habitat with human beings and transfer to humans, causing infection on exposure. Rainfall may cause exposure to *Legionella* infections and lead to the corresponding disease called Legionellosis [72]. Multiple studies have been devoted to infections related to contaminated water [73]. Similarly, drought can aggravate the effluent concentration runoff, pH and chemical quality. Contamination of surface water puts treatment plants at risk, leading to poor drinking water quality, which is especially detrimental for the elderly [74]. Likewise, rainfall and floods may increase waterborne diseases. A study conducted in Vietnam linked the impact of floods to dengue, pink fever, skin problems like dermatitis, and related psychological impacts [75].

According to the WHO, “Emerging pathogens are defined as pathogens seemed to have existence in a human population for the first time, or previously but are growing in frequency into areas where they have not been reported previously, generally over the last 20 years” [76]. According to this criterion, 96 genera containing 175 species are considered to be emerging pathogens. Other than common waterborne pathogens, Helminths, *Giardia lamblia, Entamoeba histolytica*, *Legionella, Cryptosporidium, H. pylori, E. coli* O157 and viruses like norovirus, hepatitis E virus and rotavirus have been confirmed as emerging pathogens that may spread through water [54,55,56,57,58,59,60,61,62,63,64,65,66,67]. These pathogens spread through changes in climate such as change in rainfall and global weather pattern, and deterioration in the ozone layer along with the destruction associated with UV light [54]. Different aspects of climate change including rising sea levels, flooding, extreme rainfall and rising temperature have previously been assessed in terms of their transmission and spread of water-borne diseases such as cholera and malaria [77].

In developing countries like Pakistan, the literacy rate is low, especially in rural areas, and people have no awareness about water quality, waterborne diseases and water pollution. People are using the same water for drinking and agriculture purposes. There is a direct relationship between education, income and awareness about water pollution, waterborne diseases and health impacts. According to a survey, individuals with higher levels of education are well-aware of the consequences of waterborne diseases [78]. It is worth mentioning that diseases linked to the marine and water ecosystems can be caused by waterborne pathogens, as these microbes are naturally present in different settings.

**Figure 1:**
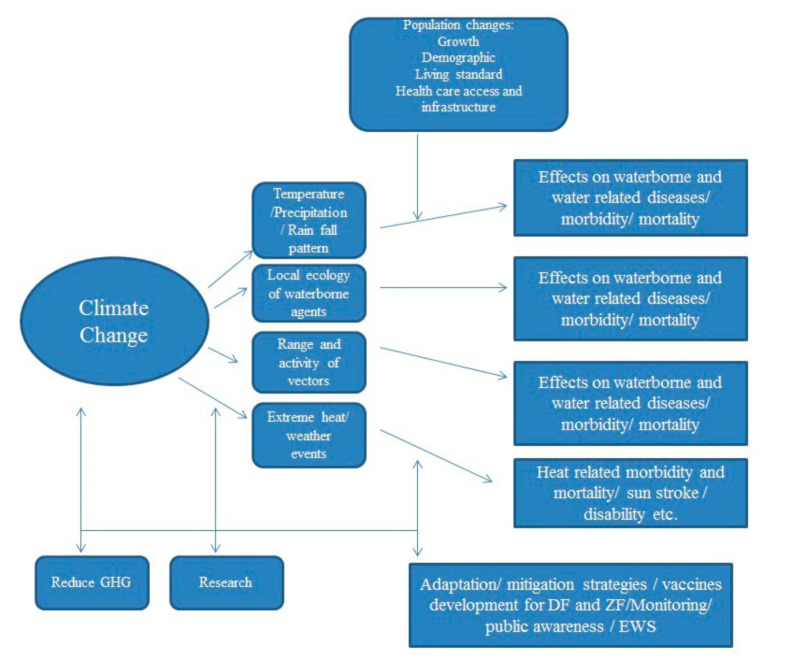
Health impacts of climate change (adapted from [79]).

This literature review shows that there is a research gap in studies that deal with waterborne diseases and climate variability, and, therefore, more research is needed to specifically explore the impacts of climate change on waterborne diseases. Figure 1 represents some of the most important factors regarding climate change-related health impacts on human beings.

### 2.5. Climate Impacts on Chemical Water Quality, Water-Related Diseases, and Health Perspectives

Climate change has significant impacts on chemical water quality when compared to changes in meteorological parameters [80]. Storm, snowmelt, drought and elevated air temperature have a significant impact on drinking water quality [81]. For instance, heavy rainfall can increase the turbidity of water resources. Similarly, an imbalance in chemical water quality has been observed due to a rise in temperature [82]. Chlorine used for decontamination of water may produce more trihalomethanes after reaction with organic acids at high temperature [83]. As stated earlier, average temperature has been increasing due to global warming, and this can impact on water resources including chemical water quality. Similarly, dissolution of chemicals, especially agriculture waste and fertilizers, can change the quality of water resources. According to Quevauviller and Umezawa [84], climate change may impact on water chemistry and sea-level rise, so salinization may be affected, which influences the depletion of freshwater and river environments. Different factors like acidification and remobilization of contaminants in sediments due to flooding and an increase in temperature can modify pollutants in water resources, which can affect aquatic life [85]. A study conducted in the Mekong Delta on climate change impacts on water-related diseases reported that limited work has been done on the relationship of climate change impacts on water quality [86].

Due to the effects of climate change, the salinization of drinking water has introduced problems for low income countries [49]. For example, salt intrusion and related health issues are common in Bangladesh [87]. Approximately 20 million people are at risk of hypertension in Bangladesh, which is a major cause of cardiovascular diseases [88,89], since more salt in water can cause hypertension and associated diseases. A study conducted in Bangladesh using an integrated salinity flux model and hydrodynamic model reported that both salinity and intrusion length has increased in the Gorai river due to the sea-level rise [90]. A similar study investigated the effects of saline contamination in drinking water on human health hazards in Bangladesh [91]. Another study reported high levels of arsenic in surface water and 2–4 times the amount, in drinking water in Bangladesh, with respect to the average eligible standards [92]. The problem of salinity and hypertension will be exacerbated in the future among people living in coastal areas due to the high intake of sodium through drinking water [93,94].

In another study conducted in Beijing, China, post-flood water quality was reported to have quality samples unfit for drinking purposes [95]. Indeed, both floods and drought conditions deteriorate the chemical quality of water, which leads to significant health impacts and high risks for consumers (Table 3).

According to the Intergovernmental Panel on Climate Change (IPCC) Fourth Assessment Report (AR4) Climate Change [103], climate change-related amendment can affect diseases caused by water, which are categorized as waterborne, water-related, water-washed and water-based. The main considerations proposed in AR4 in order to find the relationship between climate change, water quality and water availability are below:

1. The linkage between water availability, access to improved water, and health burden due to diarrheal diseases;
2. The role of rainfall in waterborne disease outbreaks through water supply;
3. The effect of temperature both on chemical and biological water quality; and
4. The direct effect of increased temperature on diarrheal diseases.

It has been reported that climate change can affect water-related diseases like malaria, dengue fever, and other infectious diseases. According to Rogers [104], one-third of the global population lives in places linked to dengue transmission. Similarly, malaria is a rainfall-dependent disease and decreases with reductions in rainfall.

### 2.6. Elucidation to Diminish Water-Related Issues

Numerous methods and remedies have been used to control mosquito-related diseases, and the best of these is to control the existence of mosquitoes, which involves chemical, biological, environmental management, personal protective measures and physical methods [105]. Chemical methods include the use of tested and recommended insecticides, e.g., pyrethroids for killing adults and larvae. These should be used under the supervision of experts and trained staff such as a team of entomologists, a vector control supervisor and field staff [106].

Direct chemical spraying or aerial spraying of chemicals by low flying aircraft (to cover a large area or when there is limited access by vehicles) should be accomplished at the habitats, resting sites and breeding places of the target insects at regular intervals of 2–3 weeks. In-house spraying should also be done in all bedrooms, washrooms, wall corners, etc. For dengue control, man-made habitats should be screened, and Methoperene/Altosid (Briquets) and Diflubenzuron (Dimlin) should be applied.

As reported by Yi et al. [107], diesel oil is effective in killing larvae and pupae of mosquitoes in small waterbodies, but this can also kill other aquatic animals and is unsustainable. They suggested golden bear oil as an alternative, but this product is only available in the USA. They also suggested various methods to control mosquitoes using mosquito traps, genetically modified male mosquitoes and mosquito counter devices. Furthermore, indoor fogging or space spraying is an effective way to control dengue [108]. Larvicides should be applied on clean and stagnant water.

Multi-purpose environmental management of marshes, open drains, standing water in open fields, surface water, gardens and waste is required for disease control. Personal protection measures include personal protective clothing, bed nets (long lasting insecticide treated nets and curtains at doors), use of gauze on doors, and insect repellent lotions. Picaridin/Icaradine and N,N-diethyl-meta-toluamide (also called DEET) are recommended repellents that can be used in emergency cases. Cloth can be treated with permethrin to control mosquitoes, at the recommended dose of 1.25 mg/m^2^ after every five washes. Even simple physical methods such as closing doors, especially in the morning and evening, have a positive impact on preventing diseases. Rapid population growth and urbanization, especially encroachments, provide ideal places for breeding of mosquitoes. In the absence of medicine and therapy, it is better to control this growth and breeding of mosquitoes and other vector-spreading microbes [109,110,111].

Concerning the environmental consequences of changing climate, more attention is required from experts, authorities and health departments on preventing the spreading of lethal diseases such as dengue and malaria. It is advisable that malaria and dengue control programs should be a part of national health policy with strong resource commitment and implementation. Increasing awareness and educating society is a vital element to cope with spreading of waterborne diseases (e.g., dengue fever). These programs can be started by educational institutions, offices, meetings, community reunions, etc. Besides, cleaning at household level with detergents, insecticides and other surface cleaning agents is highly recommended. Media can also play an important role in enhancing awareness through newspapers, TV programs, talk shows, etc. Likewise, a reduction of breeding sources of mosquitos and the introduction of waste management campaigns are important at community level. Indeed, health protection campaigns should be the top priority.

According to the literature, people in South Africa spent about eight hours daily in fetching water and only 19% treat their water before use. Government subsidies on water treatment chemicals and fuels for boiling water may help in increasing the percentage of people treating their drinking water and reducing waterborne diseases [112]. Regarding improving water quality, both adaptation and mitigation measures are required. In this respect, infrastructure improvements, reduction of pipe leakage, introduction of advanced water purification systems, and direct supply of clean water are necessary for the provision of safe drinking water [82]. During periods of flooding, water treatment is of great importance in controlling waterborne diseases [113]. Other interventions and home water treatments including chlorination and UV treatment [114]. There is a strong need to establish new sustainable development policies to preserve water. Without inaugurating new policies, around 40% of the world’s population is projected to experience severe water stress by 2050, especially in Africa and Asia, where the population is projected to increase from 7 billion to over 9 billion by 2050 [115].

## 3. Pakistan’s Perspective, the Status Quo

### 3.1. Water Quality Issues

Based on the long-term CRI, Pakistan was the fifth most affected country in the world during the period between 1999 and 2018 [116]. Moreover, Pakistan severely suffers from water shortage and lack of clean drinking water [85]. In general, just 20% of the country’s residents have access to clean potable water, which makes the remaining 80% dependent on polluted and unhealthy drinking water [117,118]. Many empirical studies have been conducted on water quality issues in Pakistan, but some important studies on biological and chemical water quality conducted in different cities across all the provinces of Pakistan have reported on the deterioration of water quality throughout Pakistan and highlighted an increase in waterborne bacterial and other related diseases (Table 4). The lack of access to safe drinking water causes waterborne diseases, which constitute about 33% of all deaths [118]. Another study reported that between 20% and 40% of all diseases in Pakistan are due to poor quality of water [119]. This can be explained by deficiencies in waste management, lack of protection of water resources, poor sanitation, adverse anthropogenic activities and lack of social awareness [120]. A general analysis of water quality data indicates the poor circumstances of water resources in Pakistan (Table 4), highlighting the need for new water treatment policies. Roughly 60 million Pakistani residents are affected by high levels of arsenic in their drinking water [121]. Rural areas are more vulnerable in terms of access to safe drinking water compared to major cities or the capital city. A study of the Tehsil of Jehlum district found more than 80% contaminated water [122]. Even water supplied to schools was poor in terms of drinking quality [123]. It is worth noting that Pakistan mainly relies on the Indus River as one of the main surface water resources. However, climate change has been negatively impacting on the Indus River, which has increased the pressure on sustainable water resources [124]. A 50% reduction of the flow rate of the Indus River would have a detrimental impact on public health, environmental protection and public finances [125]. Similar consequences can be envisaged for other developing countries like Ethiopia, where major rivers have faced decreases in both water quality and quantity [126].

Clean and healthy drinking water has a high impact on recreational activities, fisheries, tourism and sports. However, potable water resources can become polluted, which negatively impacts on both economic and health aspects [126]. According to reports by the Pakistan Council of Research in Water Resources, a survey was conducted in 23 major cities of Pakistan; four major contaminants prevailed in Pakistan; most contaminants were of bacterial nature (69%). This was followed by arsenic (24%), nitrate (14%) and fluoride (5%) [167]. According to the report, 69% of sources were contaminated according to the National Standards for Drinking Water Quality. According to a Khyber Pakhtunkhwa (KP) health survey, in 2017 89% of households had access to improved drinking water. This is similar to the 94% figure regarding Punjab province as reported by the Punjab Government [168]. Efforts have been made by the Punjab Government to provide clean and contaminant-free water. For example, some important projects including the Punjab Saaf Pani (PSP) project, worth 70 billion PKR (1 US $ = 158 PKR), have been launched to provide clean drinking water to poor urban and rural areas. For 2015–2016, 11 billion PKR were allocated for medium-term development goals. The PSP is designed to provide 3 L of clean drinking water per capita as part of the approved plan. The program promotes the installation of filtration plants, new water supply schemes and rehabilitation of existing schemes. Water treatment plants have been installed in Bahawalpur, Bahawalnagar, Lodhran and Rahimyar to supply safe and clean water to these cities.

Pakistan’s gross domestic product in 2018 was 314.6 billion US $. A project entitled “Changa Pani Programme” was launched to maintain sanitation schemes and provide rural water supply. A total of PKR 1 billion have been allocated for this program. Sustainable operation and maintenance mechanisms of rural water supply schemes are another initiative running in Punjab. Under this scheme, 199 dysfunctional water supply systems have been identified, while an initiative has been taken to rehabilitate 135 rural water supply schemes in Rajanpur, Chakwal, Vehari and DG Khan with the assistance of UNICEF. Similarly, in the 2020–2021 budget, PKR 6 billion were spent on clean drinking water (Punjab Aab-e-Pak Authority) and PKR 3.29 billion on water supply and sanitation [169]. For KP, 18.6 PKR billion were invested in the water sector [170]. For Sindh province, PKR 19.3 billion were spent on water supply and sanitation, while PKR 39 billion were invested on water supply and sanitation schemes including 398 projects in 2019–2020. PKR 1.94 billion were spent by Karachi city [171]. For Azad Jammu and Kashmir (AJK), PKR 700 million were invested on water use charges schemes and PKR 540 million on none-specified water categories. Similarly, GB and Balochistan did not specify water investments, but overall allocations for development work have been recorded. More initiatives and fair use of budgets for clean drinking water and water supply schemes are required in other provinces of Pakistan to fulfill the demand for clean drinking water, and to reduce waterborne diseases.

No specified data have been found on waterborne diseases in hospitals. However, dengue-related data are available, as surveillance teams of public health departments along with the government are monitoring dengue-related cases. It is highly recommended that patients are registered as suffering from, for example, gastroenteritis or shigellosis for proper monitoring at the national level. Typhoid, abdominal cramps and diarrhea are the most common water- and food-related illnesses; the number of patients varies from district to district in each province, but without registration it is very difficult to find and distinguish patients suffering from different specific diseases.

### 3.2. Water Governance and Sustainability

Water availability and linked water quality are being heavily impacted upon by climate change throughout the world, especially in Pakistan. Changes in rainfall patterns, shifting of seasons, increase in temperature, droughts, heatwaves and storms are affecting water resources. Demand for water is increasing due to an increase in population, urbanization and industrialization. It is important to manage the existing water resources. In order to achieve Sustainable Development Goal 6, ensuring availability and sustainable management of water sanitation for all, water governance is essential.

Water governance is concerned with the social, economic, administrative and political organization that influences the use of water and its management. It is important to discuss the management of water, rights to water, service provider roles and allied beneficiaries. Water governance discusses the formulation and implementation of water policies, legislation, the role of institutions, civil society and the general public in relation to provision of services and water usage.

A Pakistani national water policy has been approved in April 2018 and the water act has been implemented in almost all provinces except Sindh Province. Lack of coordination among the institutions as well as capacity building and funding constraints are important challenges to be addressed. Equity and social balance are important in addressing water governance-related issues. There are opportunities to address these issues with, for example, IT-based monitoring systems for dealing with accountability and water theft. Public–private partnerships are important in tackling water-related challenges. A good example is the water metering and pricing program of Bhalwal City in the Sargodha District of Punjab Province, where authorities have successfully implemented 24/7 supply of safe drinking water. Similarly, smart water metering has been installed in one of the sectors, named I-8, of Islamabad for the said initiative. (In Islamabad, different sectors are named alphabetically). International collaboration can help in capacity building and knowledge sharing. Awareness regarding water conservation and strategies to conserve water at all levels is necessary to save water. The inclusion of information on climate change and water conservation in the educational curricula at all levels is recommended. Fines should be imposed on violators and incentives should be given to the general public by the water authorities for water conservation and for following water laws. These kinds of initiative can help in water governance and sustainability in the future.

## 4. Conclusions and Recommendations

This literature review indicates that global warming has led to an increase in the average temperature around the globe, which has been heavily impacting on water resources, especially in Africa and Asia, as agriculture is mostly dependent on river water flow. Several developing Asian countries have already encountered the consequences of water stress. Hence, river water monitoring is an essential requirement, especially due to the impacts of climate change such as glacier melting, rainstorms and droughts.

Increases in population and anthropogenic activities have heavily influenced water resources and increased water pollution. Indeed, various studies have reported that water pollution has increased in the last decades, and consequently water-related diseases influence the health of many citizens in developing countries. The following are important recommendations which can be helpful in coping with the consequences of climate change in terms of water-related challenges:

- Due to the shift in seasons, in some locations as a result of climate variability, new water resources (e.g., melting glaciers) have been emerging. However, there is a need to manage and store water for present and future use. For instance, watershed management with dam systems might alleviate drought and floods.
- Developing effective treatment methods e.g., [172,173,174], for addressing the sixth United Nations sustainable development goal, which deals with fecal contamination (69% fecal pollution has been reported in 23 major cities) and provision of safe drinking water to the general public.
- Adaptation strategies such as protection of water resources and watershed management should be adopted to cope with unforeseen situations and to decrease the water-related disease burden.
- Education and social awareness play a major role in confronting and controlling water pollution, waterborne, and water-related diseases, and subsequently in improving human health in developing countries.

These recommendations are also valid for many other countries with similar challenges to Pakistan.

## Figures and Tables

**Table 1:** The list of the top 10 countries most affected in the Climate Risk Index (CRI; annual averages; adopted from [14]) between 1995 and 2014.

CRI 1995–2014 (1994–2013)	Country	CRI Score	Annual Death Toll Average	Annual Death Average/100,000 Inhabitants	Total Losses (Million US$)	Losses/Unit GDP in%	Number of Events (1995–2014)
1 (1)	Honduras	11.33	303	4.41	570.35	2.23	73
2 (2)	Myanmar	14.17	7137	14.75	1140.29	0.74	41
3(3)	Haiti	17.83	253	2.76	223.29	1.55	63
4(5)	Philippines	19.00	927	1.10	2757.30	0.68	337
5(4)	Nicaragua	19.00	162	2.97	227.18	1.23	51
6(6)	Bangladesh	22.67	726	0.52	2438.33	0.86	222
7(7)	Vietnam	27.17	361	0.44	2205.98	0.70	225
8(10)	Pakistan	31.17	487	0.32	3931.40	0.70	143
9(11)	Thailand	32.33	164	0.25	7480.76	1.05	217
10(9)	Guatemala	32.50	83	0.66	407.76	0.50	88

**Table 2:** Eight most populous countries in 2016 and their prospective population by 2050 (adapted from [44,46]).

S#	Country	Population in 2016 (Million)	Population in 2050 (Million)	Difference (Million)	Variation (%)
1	China	1378	1344	−34	−2.47
2	India	1329	1708	379	28.51
3	United States	324	398	74	22.83
4	Indonesia	259	360	101	38.99
5	Brazil	206	226	20	9.71
6	Pakistan	203	344	141	69.45
7	Nigeria	187	398	211	112.83
8	Bangladesh	168	202	34	20.23
	Total	4054	4980	926	22.84

**Table 3:** Potential health impacts of major physico-chemical contaminants in developing countries including Pakistan.

Chemical Contaminant	Associated Health Risk	References
Arsenic	Skin cancer, lung cancer and other internal cancers	[96]
Lead	Can cause serious damage to brain, kidneys and the peripheral nervous system.	[97]
Nitrate	Methemoglobenamia in infants	[98]
Copper	Nausea, abdominal pain and vomiting	[99]
Fluoride	Physiological disorders, skeletal and dental fluorosis, thyroxine changes and kidney damage	[100]
Chloride	High levels in drinking water may affect acceptability of drinking-water	[101,102]
Sulfate	Can produce laxative effects at high levels and effects acceptability of water	[102]
Sodium/Salinity	Hypertension, pre-eclampsia and eclampsia as well as cardiovascular diseases and related health problems	[49,87,88,89]

**Table 4:** Water quality situation in different provinces of Pakistan and associated impacts on the parameters studied.

Province	Key Parameters Studied	Water Quality Assessment and Impact Summary	Actions by Government	Improvements Required	References
Islamabad Capital Territory (ICT)	Bacteriological study by membrane filters; bottled water analysis; and filtration plant drinking water analysis.	Major waterborne pathogens identified. Bottled water has parameters below the National Environmental Quality and WHO limits. Presence of *Escherichia coli*. Filtration is not efficient to remove contaminants and water remains unfit for human consumption.	Bottled water is regularly monitored by the government. Surveys are performed by NGO and researchers.	Need to identify sources of contamination, and fines should be imposed by the authorities to bottled water companies. Replacement of cartridges.	[126,127,128,129,130,131,132,133,134]
Punjab	Bacteriological study by membrane filters; bacteriological analysis of drinking water of hospitals and households; arsenic and water quality; and multi-stage sampling technique for deteriorating water quality impacts on females.	Major water borne pathogens identified. Analyses were done both in summer and spring with high contamination results obtained during summers correlating with the growth of bacteria at high pH and temperature. The areas with low socio-economic status possessed maximum contamination (43.6%) as compared to areas with medium and high socioeconomic conditions showing 36.5% and 22.9% contamination, respectively. Entering of raw sewage into the damaged water supply network. Increased arsenic concentration in groundwater. Major waterborne diseases and profound impacts on health outcomes. Microbial contamination by *Eschrichia coli* and coliforms in general were found in water samples from Vehari in the Punjab region.	Water quality surveys are performed by the health department, NGO and researchers. Cartridges are installed in major cities to support general public water supply schemes; Changa Pani program was initiated by the Punjab Government and by previous government. 10% budget increase for 2020 as compared to previous years (2018–2019). for existing and new schemes.	Need to identify contamination sources after performing surveys. Upgrading of poor infrastructure and replacement of cartridges when and where required. One cartridge in an area is not enough, especially in summer to fulfil demand. Improvement in drainage system and new schemes to decrease fecal contamination.	[78,135,136,137,138,139,140,141,142]
Sindh	Employed membrane filtration method to assess bacteriological water quality. Physio-chemical and bacteriological assessment of drinking water by using the Water Quality Index.	Municipal water was contaminated with fecal pollutants and bacteria including different levels of resistance to tested antibiotics. Major waterborne pathogens identified. Groundwater contamination in Sujawal district. All samples showed presence of *E. coli* and fecal coliform bacteria. Phycio-chemical parameters were below national standards. Higher bacterial contamination was attributed to seepage of wastewater into drinking water networks and absence of chlorine residuals in any of the samples. In some regions such as Gadap and Kathore, roughly 30% of people have been found infected with viral hepatitis. Available water is contaminated with chemicals, pathogens and toxins.	Water quality surveys are performed by the health department, NGO and researchers. NGO are installing cartridges to fulfill the demand. Poor or intermittent water supply.	Home purification methods require further refinement and evaluations. Need to identify contamination sources after performing surveys. Upgrading of poor infrastructure. New water supply schemes, especially in the rural areas should be introduced	[124,143,144,145,146,147]
Khyber Pakhtunkhwa (KP)	Microbiological quality assessment of drinking water by the most probable number technique; correlation between poor quality of drinking water and various waterborne diseases. Regression model applied on various stream quality parameters. Physicochemical drinking water quality. Bacteriological study using membrane filtration techniques. Water quality risk assessment of surface and groundwater resources. Physio-chemical and bacteriological assessment of drinking water. Arsenic in drinking water. Post flooding study of drinking water quality.	Fecal coliforms were detected in 37% of samples, while 18% of samples were contaminated with *E. coli.* Drinking water was found to be heavily contaminated with *E. coli*, *Enterobacter*, *Salmonella* and *Clostridium*. The analyses revealed that water at most locations was not fit for drinking. Major waterborne pathogens identified showing poor quality of drinking water. About 31% diarrhea rates among children with non-improved sanitation facilities. The contamination was attributed to improper sanitation practices. The water was recommended for treatment before use. All samples showed the presence of *E. coli* and fecal coliform bacteria, but the phycio-chemical parameters were below national standards. Coliforms were found in samples together with elevated concentrations of lead, cadmium and nickel. About 67% of water samples were found to be contaminated with fecal and total coliforms. Contamination of water samples collected from villages. Drinking water is heavily contaminated with arsenic in areas of Peshawar city. Heavy metal pollution along with high electric conductivity and turbidity values.	Water quality surveys are performed by the health department, NGO and researchers. NGO are installing cartridges to fulfill the demand. Poor or intermittent water supply.	Installation of cartridges are recommended. Improvement of water supply infrastructure. Sources of contamination should be identified and rectified for contaminant-free supply of water. Improvements in water storage habits and drainage system. New schemes to decrease fecal contamination. Variations in the different districts of KP.	[148,149,150,151,152,153,154,155,156,157,158,159,160]
Gilgit Baltistan (GB)	Assessing physical, microbiological and chemical quality of drinking water.	The water was found to be highly contaminated with thermophilic coliforms throughout the year. No contamination at source, but problems for end-users. Heavy metal pollution along with high electric conductivity and turbidity values.	Water quality surveys are performed by the health department, NGO and researchers. Water supply scheme for safe water supply.	Regular monitoring and replacement of cartridges for contaminant-free water supply is recommended.	[160,161,162,163]
Azad Jammu and Kashmir (AJK)	Bacteriological study using membrane filtration technology. Study of earthquake-effected areas. Evaluation of the heavy metal status in drinking water.	Major waterborne pathogens identified showing poor quality of drinking water. About 69% of available water was contaminated by *E. coli.* Overall, 66% of water samples were acceptable; the remaining samples had heavy metal contamination surpassing permissible limits. 71% of samples were contaminated at household level. 33% samples were contaminated with heavy metals.	Water quality surveys are performed by the health department, NGO and researchers.	Installation of cartridges is recommended	[121,164]
Balochistan	Fluoride content. Bacteriological analysis. Hydrochemistry.	90 of 150 water samples were found unfit for consumption. Risk of mild to severe dental fluorosis. Total and fecal coliforms were analyzed. Unfit sample proportions: Loralai (91%), Khuzdar (91%), Quetta (76%) and Ziarat (100%). Physicochemical parameters were above the permissible limits of WHO standards.	Water act approved in 1978. The government (Pakistan Council of Research in Water Resources - PCRWR) is monitoring water quality.	Implementation of policies. Improvements in terms of water availability and quality are recommended.	[165,166]
